# Migrants’ and refugees’ health status and healthcare in Europe: a scoping literature review

**DOI:** 10.1186/s12889-020-08749-8

**Published:** 2020-06-30

**Authors:** Adele Lebano, Sarah Hamed, Hannah Bradby, Alejandro Gil-Salmerón, Estrella Durá-Ferrandis, Jorge Garcés-Ferrer, Fabienne Azzedine, Elena Riza, Pania Karnaki, Dina Zota, Athena Linos

**Affiliations:** 1 Uppsala University, Department of Sociology, English Park Campus - Centre for the humanities, Thunbergsvägen. 3H, Uppsala, Sweden; 2 University of Edinburgh, School of Social and Political Science, Chrystal Macmillan Building15a, George Square, Edinburgh, EH8 9LD UK; 3 Polibienestar Research Institute, University of Valencia, Carrer del Serpis, 29, 46022 València, Spain; 4 Arènes and ICM fellow, Rennes, France; 5 Department of Hygiene, Epidemiology and Medical Statistics Medical School, National and Kapodistrian University of Athens, 75 Mikras Asias, Goudi, 11527 Athens, Greece; 6 Prolepsis, Institute of Preventive Medicine Environmental & Occupational Health 7, Fragoklisias street, 151 25 Marousi, Greece

**Keywords:** Migration, Healthcare, Refugees, Asylum seekers, Services, Policy, Vulnerable migrants, Social care, Equality, Discrimination

## Abstract

**Background:**

There is increasing attention paid to the arrival of migrants from outwith the EU region to the European countries. Healthcare that is universally and equably accessible needs to be provided for these migrants throughout the range of national contexts and in response to complex and evolving individual needs. It is important to look at the evidence available on provision and access to healthcare for migrants to identify barriers to accessing healthcare and better plan necessary changes.

**Methods:**

This review scoped 77 papers from nine European countries (Austria, Cyprus, France, Germany, Greece, Italy, Malta, Spain, and Sweden) in English and in country-specific languages in order to provide an overview of migrants’ access to healthcare. The review aims at identifying what is known about access to healthcare as well as healthcare use of migrants and refugees in the EU member states. The evidence included documents from 2011 onwards.

**Results:**

The literature reviewed confirms that despite the aspiration to ensure equality of access to healthcare, there is evidence of persistent inequalities between migrants and non-migrants in access to healthcare services. The evidence shows unmet healthcare needs, especially when it comes to mental and dental health as well as the existence of legal barriers in accessing healthcare. Language and communication barriers, overuse of emergency services and underuse of primary healthcare services as well as discrimination are described.

**Conclusions:**

The European situation concerning migrants’ and refugees’ health status and access to healthcare is heterogeneous and it is difficult to compare and draw any firm conclusions due to the scant evidence. Different diseases are prioritised by different countries, although these priorities do not always correspond to the expressed needs or priorities of the migrants. Mental healthcare, preventive care (immunization) and long-term care in the presence of a growing migrant older population are identified as priorities that deserve greater attention. There is a need to improve the existing data on migrants’ health status, needs and access to healthcare to be able to tailor care to the needs of migrants. To conduct research that highlights migrants’ own views on their health and barriers to access to healthcare is key.

## Background

The European Union (EU) comprises a heterogeneous population that includes migrants coming from non-European countries. A migrant is here intended, according to the UN definition, as “someone who changes his or her country of usual residence, irrespective of the reason for migration or legal status” [1]. According to Eurostat data, on 1st January 2014, there were 33.5 million people born outside the EU, which represents 6.6% of the total EU population. Of these, 19.6 million were still citizens of countries outside the EU, while 14.3 million were citizens of one EU country, but living in another one [2]. In 2015 the EU received more than 1.2 million first time asylum applications. Although this is a much smaller number compared to migration within the global South, it is still more than double the number received in the previous year, which has raised both interest and concern around the impact of migrants and refugees on European healthcare systems. The number of new asylum applications has decreased since 2015 to fewer than 600,000 in 2018, yet the concerns have not been eased. Migrants from different European and non-European countries imply new demands on national public services, not least healthcare. Such demands are going to be the rule, as the fast altering political situations in various countries around the world, the ongoing global financial crisis, together with the growing implications of climate change makes it likely that a new migration wave may happen even when restrictions are put in place by EU member states.

All the EU member states have formally recognised the right for every person to the highest attainable standard of physical and mental health. However, due to the variations in socioeconomic level in the various European Union states, the different healthcare systems as well as the variation in the number of migrants arriving, achieving data to describe the situation, let alone achieving this highest attainable standard across EU countries is challenging. Recording the citizenship of legally recognised migrants captures part of the population of immigrants, but does not include naturalised migrants. Numbers of irregular or undocumented migrants are difficult to ascertain and definitions differ by member state within the European Union and across countries, making comparisons challenging. How healthcare can and should be provided for national populations with high levels of immigration is a crucial issue, but one that is difficult to address even if good quality data were available. Some attempts have been made to bring attention to these data gaps, including a report [3] providing an overview of migrants’ health in Europe, documenting knowledge gaps and calling for action, and a study [4], which looked at various issues in regards to migrants’ health needs globally, showing the discrepancy that exists between emphasis on health rights and equity on the one hand and the actual provision of equal healthcare on the other. Considering the importance of providing optimal healthcare for migrants, it is necessary to gain an overview of migrants’ access to healthcare in various European countries. This review provides an overarching picture of the current state of knowledge regarding migrants’ health status, access to and use of healthcare in Europe. This article is a scoping review that is part of the MigHealthCare project and includes Austria, Cyprus, France, Germany, Greece, Italy, Malta, Spain, Sweden— the European countries that make up the MigHealthCare consortium.^1^

The research questions guiding the review of the literature are as follows:

- What is known about the physical and mental health status of migrants and refugees in the EU member states? This topic was included in the literature review because it provides insight into the access to healthcare question.
- What is known about healthcare access and use of migrants and refugees in the EU member states?

## Methods

### Sources of evidence

Due to the scattered and fragmented nature of the literature, conducting a systematic review was not possible. Further, the topic cuts across many disciplines and methodologies and is relatively underexplored. The aim of this review was to map existing research on the topic, both qualitative and quantitative, scholarly articles and grey literature. The importance of mapping the existing research is to gain an overall comprehensive insight into what has been conducted as well as gaps in research. Given the nature of the task, and the state of the research, a scoping review is appropriate as it aims to map rather than the assess the quality of the studies that are included [5].

A scoping review of the academic and grey literature in different European languages (English, French, German, Greek, Italian, Maltese Spanish, and Swedish) was undertaken by the consortium partners.

The search was conducted according to the following main criteria specifying time-frame, databases and search terms:

- Time frame: 2011 to 2017 (the rationale for this is that 2011 saw the beginning of the Syrian and Libyan revolutions accompanied by an increase in the flow of migrants towards the EU 28 countries)
- Databases: PubMed, ScienceDirect, Scopus, PsycInfo, Social Services Abstracts (also in ProQuest), Cochrane library, CABI. Eurostat, OECD, Eurofound, CORDIS and any other databases that are available in the different languages included in the search.
- The search terms: Topic: (migrant* or migration or immigrant* or foreign* or (minority near groups) or refuge* or asylum) AND TOPIC: service* or access* or planning or delivery) AND TOPIC: (health or medical), dental care, obstetrics/gynaecology, mental health, social care

### Selection process

The evidence collected included:

- Academic articles and literature reviews (each country in its own language)
- Grey literature (think tanks, non-governmental organisation and government reports)

Articles were included in the review if they were relevant to the research questions and were in the following languages: English, French, German, Greek, Italian, Maltese Spanish, and Swedish.

### Data extraction and coding

Seventy seven papers were included in this review (see Table 1 in the Annex). The initial screening of the sources was done by the national teams. Each team used a common template to write a review in English of each source included. Uppsala University in collaboration with the national teams conducted the analysis. The sources included were coded thematically and classified according to major themes and subthemes:

- Migrants’ health status. This theme is divided in
  - ◦ communicable and non-communicable diseases
  - ◦ mental health in adult refugees and migrants
  - ◦ children’s health status
- Social determinants of health. This theme is included for the link between socio-economic condition and health status.
- Access to healthcare. The theme is divided in
  - ◦ access to maternal health services
  - ◦ communication and information issues
- Use of healthcare service
- Challenge to healthcare provision in transfer countries. This is a theme in its own right because transfer countries are reported to face common problems but to a higher degree.

This final classification was discussed and validated by the MigHealthCare consortium members.

## Results

**Fig. 1:**
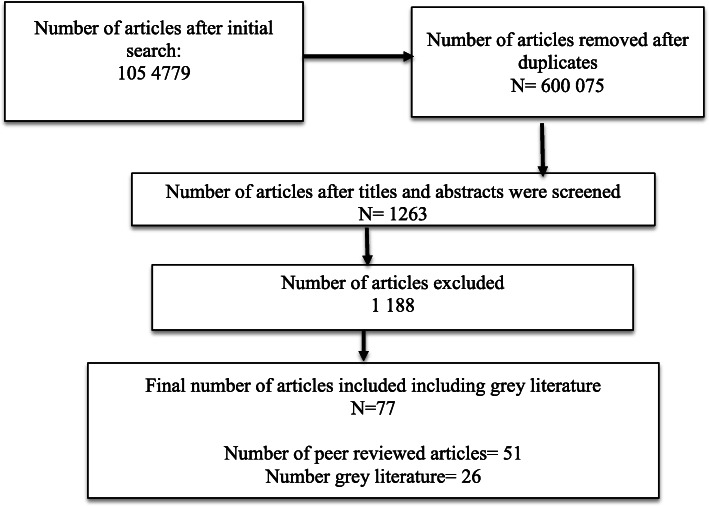
Study selection process

Seventy seven sources are analysed in this section and organised according to the above described thematic classification. Figure 1 describes the selection process.

### Migrants’ health status

The literature reviewed on health status was organized into the following focus areas: communicable and non-communicable diseases; mental health in adult refugees and migrants; children’s health status.

#### Communicable and non-communicable diseases

Migrants’ health status is influenced by the hardships of the migration process which negatively affects the physical health status of migrants. This was shown in a French study [6] that demonstrated how migrants’ health status tended to deteriorate with duration of stay which may be due to discrimination; poor employment conditions; differences in access and use of healthcare services. The study suggested that “migrant health deficit effect” (in comparison with natives), is less pronounced for second-generation migrants (people born in France to foreign-born parents). Similarly, an Austrian study showed that the population of migrant origins suffers to a greater extent than the resident population from heart disease, allergies, digestive and urogenital and dermatological problems and emphasizes the link between migrants’ health conditions and the stressful situations they face in both the workplace and the community at large [69]. Another study comparing European countries also suggested that migrants are more vulnerable to communicable diseases, occupational diseases, poor mental health, injuries, diabetes mellitus, and maternal and child health problems [8]. Two studies focusing on vulnerable migrants living in open centres in Malta [70] and in detention centres in Greece and Malta [10] showed that the prevalence of HIV, tuberculosis and non-communicable diseases (e.g. hypertension and diabetes) is higher among the non-European migrant population. However, it is difficult to compare across countries, as studies focused on specific health conditions and ethnic groups. For example, of the studies included in this review, four focused on specific conditions: ophthalmic disease [11]; HIV [13]; tuberculosis [14], skin diseases gynaecological concerns and other unattended health-related problems [15–17, 71, 72].

To conclude, data on migrants’ physical health status are insufficient on the basis of the literature reviewed. Some migrants might be at particular risk of non-communicable diseases arising from obesity and insufficient physical activity due to patterns of disease in countries of origin, disadvantageous living conditions, precarious employment and trauma.

#### Mental health in adult refugees and migrants

The articles that were reviewed show that refugees and migrants tend to have higher prevalence of mental distress compared to non-refugees in Europe. Ten of the papers that were reviewed focus on the mental health of migrants, mostly refugees. A German study found an association between depressive symptoms and migration status in the older migrant populations [73]. A more recent study described the mental health condition of asylum seekers who passed through Médecins sans Frontières clinics in Sicily between October 2014 and December 2015 and, when invited, presented themselves for mental health screening [74]. Of the 385 who were screened, most were young men who had left their home countries in West Africa more than a year prior to arrival. The most common mental health conditions were post-traumatic stress disorder (31%) and depression (20%). Most of the potentially traumatic events were reported to have been experienced in the home country (60%) and during the journey (89%), but the trauma of being a refugee was also reported, with activity deprivation, worries about people who were left behind, loneliness and fears of being sent back [74].

Another study found a connection between psychosis and a background as an irregular migrant [18]. Similarly, an Italian report underlined that although empirical data and scientific research on the topic of migrants’ mental health is still rare, practitioners and sector operators have experienced the increase in requests for psychiatric care from migrants who have lived traumatic situations, social marginalization, lack of social support and are therefore at higher risk of post-traumatic stress disorders [19]. The same Italian study highlighted how structural barriers impede the effective transfer of patients to further care facilities. High levels of stress in detention centres are linked to the reporting of non-specific physical symptoms [70] as a form of somatization of psychosocial stress suggesting underlying mental disorders.

Not only previous traumatic experience influences mental health but also duration of stay, as suggested by a study conducted by the Jesuit Refugee Service (JRS) in 2010. This study stated that 80% of Asylum Seekers interviewed reported a deterioration in their mental health since their arrival in the detention centre. From a population of around 500 detainees, 74 individuals required in-patient psychiatric care [20]. A project conducted in Malta by Aditus and a UN agency underlined mental health problems affecting a large proportion of the refugee community, including post-traumatic stress disorder, depression, anxiety, psychosis, paranoia and self-harm; feelings of isolation and loneliness were also mentioned by refugees as major concerns to service providers [41] Reported symptoms such as stress, anxiety disorders, panic attacks, and other psychiatric problems were said to be the most common symptoms in some camps more than in others [18].

There seems to be a growing interest in the mental health condition of migrants, even though the studies that look at this issue seem to focus mostly on refugees— for whom there is a presumption that mental health problems arise from uncertain migration status.

#### Children’s health status

Five studies focused specifically on children’s health status. A French study showed that children born in a hepatitis A endemic area have a significantly higher prevalence of hepatitis A seropositivity compared to children born in France, possibly as a result of exposure during overseas trips to visit family or of family members visiting from the endemic areas, implying an urgent need to vaccinate children [75]. Another study in France underlined the lack of clinical practice recommendations for the care of unaccompanied refugee minors as causing significant disparities depending on the *department* or region to which the young person arrived. The most frequently diagnosed serious conditions were digestive parasites, schistosomiasis, filariasis, hepatitis B and iron deficiency and the failures of care implied the need for standard care to be defined [22].

Vaccination status and dental issues as well as Latent Tubercolosis Infection (LTBI), anaemia, low serum ferritin, eosinophilia, and protective antibodies among migrants were discussed in a Greek study of child migrants [23]. Reports of hypothermia after arrival by sea and mental health challenges associated with the experience of violence, separation from family, insecurity, inadequate housing, trafficking, and sexual exploitation were also recorded [24].

Oral health was also discussed in a study of 12-year-old migrants in Austria that showed the prevalence of caries among children born to migrants was 42% higher compared with children with no migrant background. Children with a migrant background were more affected by gingivitis (gum inflammation) and less likely to seek orthodontic treatment or counselling compared to other 12-year-olds. The report underlined how better use of group prophylaxis and individual healthcare prevention would be a means of reducing unequal distribution of health risk [25].

Generally, our review found that references describing child migrants’ health status are limited, country specific and focus on specific illnesses, making it difficult to draw comparisons and commonalities across countries or to determine the health status of children with migrant background within each country.

### Social determinants of health

In the WHO definition, the social determinants of health “are the condition in which people are born, grow live, work and age” and bear the major responsibility for differences in health status. As it affects health status, we decided to include the literature looking at social determinants of health in this review. The majority of studies [69] in this review looking at social determinants of health were conducted in France [6, 26, 27, 29–32] and three in Malta [21, 28, 70]. According to a systematic literature review, the link between socio demographic conditions and health is stronger for migrants than for the native population [6]. In France, studies reported on the increased health risks for homeless migrants [26], including chronic diseases. A hospital polyclinic in Paris used overwhelmingly by migrants was surveyed and, although their average duration of stay in the country was 12 years, about half of the sample were undocumented and a quarter had no health insurance. Vulnerable migrants in France (including minors, women, and people with disability) were found to have poor self-rated health and poor living conditions as well as being exposed to violence. A study focused on the health effects of violence [7], showed that 84% of 128 women migrants consulting a ‘Médecins du Monde’ clinic in Paris had faced violence, whether verbal, physical or sexual.

Prevalence of having experienced violence and insecurity was higher for people living in public emergency accommodation and those who were homeless than for those living in camps or in someone else’s accommodation according to people’s declaration [29]. Migrants and especially women migrants from sub-Saharan Africa in France in 2012–2013 faced precarious administrative and social conditions, associated with poor health outcomes [30]. The same study showed that compared to women, men’s diagnosis occurred after a longer delay following arrival in France and occurred more frequently during an active phase of the disease. An ethnographic study from 2015 showed that the availability of accommodation for migrants was positively linked to migrant access to healthcare [30] .

Discrimination against migrants’ access to employment or healthcare was shown to be a pressing issue in another French study. Discrimination due to a migrant’s country of origin is shown to have consequences for health status not just for immigrants who are newly arrived, but throughout their life course [32].

In a study from Malta the social and economic environment of migrants, the harsh living conditions in open centres and detention homes, have been shown to lead to negative health outcomes [70]. Other factors such as limited access to paid work was raised as a concern in the literature that was reviewed. Reference was made to particular sub-Saharan African asylum seekers who seemed especially vulnerable to exploitation and abuse [28]. Over 2000 immigrants in Malta, living in open centres were assessed between August 2010 to June 2011 and the following factors were found to be detrimental to health outcomes: the environment in detention homes including exposure to cold, a lack of space and overcrowding, a lack of activity, poor diet and high levels of stress; together with a lack of systematic and/or preventive medical care and a lack of treatment for infections and diseases. Furthermore, the report emphasizes how the detention context poses additional significant challenges for asylum seekers and migrants with chronic medical conditions, disabilities or mental health problems.

Although these results emphasize the need for stable accommodation available regardless of the migrant’s legal status as a key means of promoting increased health equality for migrants, the studies only reflect findings from France and to some extent Malta, making it difficult to generalise.

### Access to healthcare

Evidence of migrants’ access to healthcare is scant. Access to healthcare for refugees, asylum seekers and migrants varies across European countries in terms of regulation and laws [33]. Even when legal accessibility is available, differences and inequalities still exist in accessing healthcare [12, 34, 35, 56].

Organisational and administrative issues were highlighted as barriers to access healthcare for migrants in studies from Italy and Greece [35]. A European report (2016) suggests that there is a lack of institutionalized procedures for taking care of unaccompanied minors leading to frequent breaks in the continuity of care [18].

Undocumented migrants’ access to healthcare is especially problematic [37]. Two qualitative studies [18, 40] and a narrative review [36] focused on undocumented adult migrants and refugees in different European countries, health needs and access to health services and concluded that healthcare services are underused by undocumented migrants, since these migrants tend to be unaware of their entitlement, and when they receive care, it tends to be inadequate.

Marques (2012) reviewed countries in regard to access to healthcare for undocumented migrants and refugees showing a multi-faceted picture. Even though access to healthcare may be granted by law, as in France, other barriers such as lack of knowledge, administrative requirements, language difficulties, and fear of being reported, as well as discriminatory practices and refusal of care are mentioned as obstacles to accessing care [42].

In the section below, we review communication and information issues and particular factors affecting access to healthcare for migrant women.

#### Access to maternal health services

Evidence on maternal healthcare focused mainly on specific issues such as female genital circumcision (FGC) [76], the delayed use of maternal health services by certain groups of migrants [48], and inequalities in pregnancy and childbirth [49]. FGC was investigated together with prenatal care in refugee women from Syria, Somalia, Libya, Eritrea, Ethiopia, and the Ivory Coast in Malta [76]. Obstacles identified in access to healthcare included language barriers, not only within the healthcare setting, but also in using transport to reach healthcare services [76].

Insufficient interpreters and lack of cultural mediators, communication and information barriers were mentioned in two studies [49, 76]. These obstacles led to women missing important appointments, required medical tests remaining incomplete and women feeling uncomfortable [76]. Existing inequalities in childbirth outcomes for migrant women in Europe were evident, and underlined the lack of evidence for planning improved care and access to care [49].

#### Communication and information issues

Evidence showed a heterogeneous situation in European countries concerning health literacy between migrants and non-migrants [51]. A number of studies highlighted under-addressed cultural and communication issues described below [44–46, 77] between migrants and healthcare providers leading to poor health service provision for migrants, governance problems and incoherent distribution of power and responsibility for the provision of healthcare between different actors as reported by an Italian study [47].

A German comparative study looked at migrants from several European countries and demonstrated that migrants make more use of first-aid stations; show predictable communication and understanding difficulties and have different views about health and illness compared to ‘non-migrants’; the results were suggestive of barriers to the use of regular healthcare services among migrants [55].

Two studies showed that lack of information regarding available care options and language barriers were among the factors contributing to migrants’ health vulnerability [38, 39]. A lack of knowledge concerning specific diseases such as HIV and AIDS and other sexually transmitted diseases was reported by a quantitative study of 600 migrants from “third countries” in Cyprus [39]. Factors such as “high cost, lacking awareness of the healthcare system, culturally insensitive services, different perceptions of illness and stigma, as well as limited language skills” were highlighted in a Finnish study among various groups of migrants (Russians, Somalis and Kurds) and were shown to contribute to an increasing perception of unmet needs [52].

A multi-method study in Austria focusing on migrants from Turkey and former Yugoslavia, compared various groups of migrants in terms of their previous experiences with healthcare, showed that different groups of migrants had the same level of health literacy as the general population [50]. On the other hand, 455 adult refugees speaking Arabic, Dari, Somali or English were surveyed in Sweden showing that the majority of these refugees had inadequate or limited health literacy, both functional and comprehensive. The study recommended that levels of health literacy should be taken into consideration in activities addressing migrants [53]. Another study in Spain also recommended action research as a way to tackle poor health among migrants [43].

Concerning the perceptions of migrants’ own health and unmet health needs, an Italian study among migrants in Spain and Italy shows that perceptions of unmet healthcare needs have increased from 2007 to 2012 among the migrant population in Italy [51]. By contrast, in Spain 2012, the native population’s perception of unmet needs also increased.

### Migrants’ healthcare use

Under this category we included studies dealing specifically with migrants’ patterns of use of healthcare services. Most of the studies of healthcare use tend to homogenise migrants and compare/oppose them to non-migrants who are also homogenised— e.g. [54, 55, 57]. These studies often highlighted the increased use of emergency room (ER) or acute care provision by migrants compared to non-migrants and the increased likelihood of visiting ER during unsocial hours as well as increased use of obstetrical and gynaecological services among migrant women [54, 55, 57]. At the same time, migrants, especially certain vulnerable groups such as first generation migrant women, are shown to use preventive screening and preventative services less than non-migrants [8, 9].

Similarly, a Spanish study with healthcare providers showed a perception that emergency service is the main access route for migrants and reported failures in the continuity of care for immigrant patients. Variations existed, however, among migrants depending on both their country of origin and the level of social integration [60].

A study of how migrants in Greece made use of the healthcare available to them, showed that compared with non-migrant patients, hospitalization rate was lower for chronic conditions but higher for accident-related diagnoses, treatments for infectious disease, and medical conditions related to depression or alcohol use (including: TB, gastritis/gastroenteritis, hepatitis, pneumonia, alcohol-related conditions, poisoning, and allergy) [57].

A study of the utilization of hospital services by the patient’s country of origin in Aragona, Spain showed that foreigners tend to use the public hospital less than the native population. However, this observation is inconclusive since the variation in prevalence of different diseases in immigrants’ countries of origin meant that the reason for using hospitals services varied by country of birth of the immigrants [58].

A review of mainly survey-based evidence of healthcare providers on the use of healthcare services among migrants showed no difference in the use of medical services by migrants compared to the native population. However, differences exist in the use of specialist care where migrants use of this type of care less [59].

A study in Vienna investigated the reasons for a reduced use of professional healthcare services even when needed, focusing on older migrants from Turkey, former Yugoslavia (Bosnia, Serbia), Poland and Iran [78]. The study evaluated the relevance of different reasons, from primary structural reasons (poverty, marginalization, discrimination), to a lack of knowledge about the care system, to insufficient ‘transcultural competences’ of the healthcare stuff. The solutions suggested aim at strengthening the “orientation towards the principles of openness, diversity and individuality” of the city of Vienna by recruiting people with migration experience/background as well as transcultural competencies.

Our review found that studies of healthcare use tend to offer simplified pictures of migrants versus locals, where both groups are taken as homogeneous. Such simplification makes it hard to reach a conclusion about the reasons behind the differences in healthcare use — whether socio-economic circumstances, health status, or the system’s lack of transparency and openness to diversity.

### Challenges to healthcare provision in transfer countries

Understanding the challenges of providing care for new migrants has had a particular focus in countries such as Greece and Spain that are entry points for arrivals to Europe. The challenge that the refugee crisis posed to national health services in transfer countries was said to have received inadequate media coverage and to be too marginal in public debate [62].

The studies underline the needs of caregivers in transfer countries, in terms of psychological support; additional financial and human resources; training courses. They underline the limited availability of diagnostic equipment, mental care services and an integrated provision of care for new migrants that allows them to easily access different services, including translation and cultural mediation.

A Greek study on “caregivers working in contemporary refugee hotspots” found that caregivers suffer from psychological stresses and sleep disturbances as well as post-traumatic stress syndrome (7% PTSD) [61]. Organisational issues faced by healthcare providers in these countries included problems of internal and external communication and coordination, cultural and language differences, inadequate funding, inadequate human resources to treat an overwhelming volume of refugees. Greece in particular was noted as one of the countries dealing with sheer numbers of refugees [63]. Other challenges faced by healthcare providers working in the front line in Greece included the limited availability of on-site diagnostic tests, electricity, and running water in camps [64].

A comparative study underlined the challenges faced by Greece, Italy and Slovenia. The study reported on insufficient training courses in transcultural competencies for health and social care professionals in Italy; staff shortages on the islands, lack of interpreters in emergency care departments, and a lack of suitable accommodation for vulnerable populations in Greece. In Slovenia the lack of funding to treat chronic non-communicable diseases was emphasised. In all three countries poor coordination between participating organisations, for example with regards to supplying food and clothing to reception and accommodation centres, was blamed for the gap between demand and supply of goods and services [66].

An increase in migrants’ requests for hospitalization and psychiatric care and deficiencies in the services that should provide mental care was reported by an Italian study [19]. In particular, the report referred to how traumatic and tragic experiences (including torture) and post- migration living difficulties contribute to post-traumatic stress disorder (PTSD). Although some special initiatives to address PTSD exist (the Protection System for Refugees and Asylum Seekers for example), the increased demand for support has proven difficult for the Italian State [19]. Serious deficiencies in the availability of cultural mediators and of expertise in migrant mental health, combined with the increased demand, placed a severe strain on the Italian provision of mental health services for migrants [65].

The availability and organisation of health assistance for migrants, refugees and asylum seekers through civil society organisations varies across European member states. The already mentioned qualitative report comparing Italy, Greece and Slovenia [64] shows that the services are centrally administered in Greece and Slovenia compared to Italy’s regional organisation. Healthcare services for migrants, refugees and asylum seekers in Italy have been provided mainly by health professionals appointed by the ministry of health while in Greece, non-voluntary organisations (NGOs) have been playing a big part in providing healthcare. Slovenia has state-appointed health professionals undertaking the work alongside NGOs [66]. The creation of a Refugees’ Health Unit in Greece offered the opportunity for healthcare providers working with a translator or cultural mediator [67]. In Spain, an Intercultural Mediation Programme for women mostly treated reproductive problems among Latin American women. The programme provided information, education and facilitated access to reproductive health services [68]. These last two examples suggest that integrated provision of care, whereby migrants can access a range of services, together with translation and cultural mediation as appropriate may represent a form of good practice.

According to the sources overviewed, transfer countries appear to face specific problems in the provision of healthcare for migrants and refugees, to a higher degree. Lack of money and of trained and stable human resources, organisational malfunctioning and poor coordination among the actors are all mentioned as factors hindering the provision of healthcare for migrants and refugees.

## Discussion

Most of the articles which were reviewed focused on the health status of migrants and refugees, looking at communicable and non-communicable diseases, mental health and children’s health status. Generally, the references describing health status of migrants are country specific and focused on specific illnesses, making it hard to draw comparisons across countries. Mental health is still relatively underexplored and studied mainly in relation to refugees— where the presumption that mental health problems arise from insecure migration status is confirmed. Sources addressing the social determinants of health emerged as another major focus of the current literature, especially in certain countries, and despite not being an initial focus of the research questions, were included for the link between socio-economic conditions and health status. Despite the aspiration to universal healthcare for all, inequalities persist in access and use to healthcare. Organisational and administrative issues, were highlighted including barriers, language and communication problems, overuse of emergency services and underuse of primary healthcare as well as structural and interpersonal dynamics biases towards migrants and refugees. Transfer countries are reported to face common problems but to a higher degree. Lack of funds and of trained and stable human resources; organisational malfunctioning and poor coordination among the different actors are all mentioned as factors hindering the provision of healthcare for migrants and refugees.

Although interest does exist in understanding health status and access to healthcare for migrants, the collection of data is fragmented and conducted in different settings and periods. This is an obstacle to monitoring and improving migrants’ health status as there is a lack of reliable, standardized and shared procedures for routine collection of health data on migrants in European member states, which represents a significant impediment to ascertaining migrant health status across Europe. Practice around the demographic classification of populations varies with the disclosure of “ethnic” information forbidden in Sweden, for example, on the grounds of anti-discrimination legislation [79]. This makes it difficult to provide a detailed picture of the health status of particular ethnic groups, which may overlap substantially with migrant groups at specific moments in time.

The lack of common definitions (i.e., definitions of migrants, non-migrants, optimal care, etc.) and clearly defined goals hinders analysis and comparisons. In reporting on practice, it is often unclear whether a migrant is from outside or within the European Union, perhaps because care providers do not always know and, given the sensitivity of migration status, it may be difficult to establish. A top down approach of evaluation of healthcare needs of migrants is mostly used. However, there is hardly any investigation of how migrants’ own, self-defined health needs can be met. Studies in different countries have different emphases making comparison difficult: some studies compare the health condition of migrants with the local population, with often contradictory results and context specific; other studies focus on health conditions of children pre-dating their migration (e.g. hepatitis, dental problems); while others consider the mental health unbalance between migrants and non-migrants. Reports mostly conducted in France underline the link between housing conditions and health.

Most articles on health status focused on communicable diseases. Less consideration is given to non-communicable diseases, preventative care and the question of equity in health and in healthcare access. Recent studies have reported a higher use of emergency services by migrants and a higher likelihood of visiting ER during unsocial hours; together with the higher use of obstetrical and gynaecological services among migrant women compared with non-migrant women. These discrepancies may signal the presence of barriers to migrants’ use of regular healthcare services. A few studies have investigated the accessibility of healthcare for migrants, testing intercultural policies aimed at helping healthcare providers meet the needs of migrants, while others have focussed on the conditions discouraging migrant from seeking care.

The body of scientific and grey literature reviewed here underlines that migrants’ health status and the possibility of health equality is affected by multiple factors that influence migrants’ ability to access healthcare. These include legal entitlement; knowledge of the health system in a new country; previous experience of healthcare; language and cultural barriers; health beliefs and attitudes; and the structure of the health system itself in the new country [33, 80]. The sources reviewed highlight similarities and differences among the European countries. Although the system of legal entitlement and the health system itself varies across European countries, there are problems in regard to knowledge of health system, language and cultural barriers and health beliefs and attitudes are common.

In the material that has been reviewed, there is clear evidence of the need to conduct research to highlight migrants’ own views of their health and on barriers to access to healthcare.

### Limitations and strengths

The literature review included nine countries across Europe. The fact that literature was gathered in the various languages of these countries contributes to the strength of this review by synthesising material that is often excluded from the evidence base. The partners were able to access literature, both peer-reviewed and grey literature, in their language, which increased the breadth of our search base and enabled the inclusion of a wider variety of reports from NGOs and official agencies, as well as getting beyond the literature available in English. On the other hand, the broadness of the topic and the scoping aim of the review could result in having missed some sources.

The literature reviewed was dominated by certain countries and by certain topics while others appeared less often, which may be due to the eyes of the reviewer as much as to the availability of data. The lack of a commonly held definition of what constitutes a migrant, an asylum seeker and a refugee and who counts as a vulnerable migrant, as well as the different sample sizes, analytical methods and the focus on specific ethnic groups, makes generalisation and drawing conclusions difficult. Measures and policies for migrants’ healthcare were particularly hard to account for without better data on the effectiveness of the measures introduced so far, both the policies addressing patients and those addressing providers.

## Conclusion

Evidence from different European countries shows that despite equitable aspirations inequalities between migrants and non-migrants in health and in access to healthcare services persist. Inequalities are the results of legal barriers in access to care for refugees and undocumented migrants, and are also due to the economic situation of migrants who may lack the means to pay for health services and / or may lack the language and cultural competency to navigate the healthcare systems and / or may be exposed to discrimination.

The European situation concerning migrants’ health status and access to healthcare is heterogeneous and it is difficult to compare and draw any firm conclusions due to the scant evidence. Different diseases are prioritised by different countries, although these priorities do not always correspond to the expressed needs or priorities of the migrants. Mental healthcare, preventive care (immunization) and long-term care in the presence of a growing migrant older population are identified as priorities that deserve greater attention. There is a need to improve the existing data on migrants’ health status, needs and access to healthcare to be able to provide optimal healthcare tailored to the needs of migrants. As migrants’ own voices were not highly present in the reviewed data, there is need to conduct research to highlight migrants’ own views on their health and barriers to access to healthcare.

## Annex

**Table 1:** List of reviewed studies (77 total)

Study	Country	Type of study		Study design			Population	Sample size
		Scholarly article	Grey literature	Quantitative		Qualitative		
				Cross sectional	Longitudinal			
Matlin et al. (2018)	Europe and beyond (region, country and province and city jurisdictional level)	Yes (literature review						
Aichberger at al. (2012)	Germany	Yes		Yes			German residents50+ born outside or who have immigrated to Germany	2890
Berchet & Jusot (2012)	France	Yes (Literature Review)						
Estrada & Lazimi (2013)	France	Yes		Yes (128)		Yes (33 interviews)	Women visiting MdM health facilities in Paris and St Denis	
Rechel at al. (2012)	European Union	Yes (Literature Review)						
Rommel et al. (2015)	Germany	Yes		Yes			Population-wide 18+	8151
Bozorgmeh et al. (2016)	Germany	Yes		Yes		Yes	Heads of all German public health authorities	389
Biffl (2003)	Austria		Yes (Conference paper)		Yes		Population wide	
Padovese et al. (2015)	Malta	Yes		Yes			Migrants men and women	2216
Kotsioni & Egidi (2013)	Malta and Greece		Yes (Report)	Yes			Migrants and asylum seekers in immigration detention facilities in Greece and Malta between 2008 and 2011	
D’Hermies and de Champs-Léger (2015)	France	Yes		Yes			Outpatients with ophthalmic issues from the free access to healthcare facilities at an Hospital in Paris	150
Lot et al. (2012)	France	Yes		Yes			Migrants with HIV, TB or Hepatisis B	National data base
Che & Antoine (2011)	France	Yes		Yes (epidemiological)			TB	National data base
Albares et al. (2012)	Spain	Yes		Yes (epidemiological)			All immigrant patients seen at the dermatology clinic between February 2005 and February 2006 in Alicante (Spain).	
Aguilar-Duran & Sánchez Martínez (2014)	Spain	Yes		Yes			All the patients diagnosed with TL in Hospital del Mar (Barcelona) between 1990 and 2009.	
Calderón Sandubete et al. (2014)	Spain	Yes (Literature review)						
Crepet et al. (2015)	Italy		Yes (NGO report)	Yes			Asylum seekers MSF clinics in Sicily	385
Simonnot et al. (2016)	Europe		Yes (NGO report)	Yes			Vulnerable migrants in 31 cities/12 countries	30,534 patients
ANCI,Caritas italiana Fondazione Mifa, Servizio centrale dello Sprar (2016)	Italy		Yes (Report)	Yes				
Taylor-East & Caruana (2014)	Malta	Yes						
Camilleri and Taylor-East (2010)	Malta	Yes		Yes			All patients newly admitted, with a diagnosis of psychosis; no exclusion criteria related to age, gender or ethnicity.	111 patients, 67 of whom were male and 44 female
Burbotte et al. (2011)	France	Yes		Yes			Children 1 to 15 years old	315
Monpierre et al. (2016)	France		Yes (Thesis)	Yes (Combination of biological tests, plus medical file of patient, hospital data and data from the place where the minor lives).			Minors	
Pavlopoulo et al. (2017)	Greece	Yes		Yes			Migrant and refugees Children 1 to 14 years old out patient clinic of a tertiary hospital	300
Giannakopoulos et al. (2016)	Greece		Yes (Lancet short note)					
Bodenwinkler et al. (2012)	Austria	Yes		Yes			Children 12 years old	Representative sample of randomly selected children attending public schools
Kaoutar et al. (2014)	France	Yes		Yes			Patients from the free access to healthcare facilities at the French National Health Service	581
Kaoutar et al. (2012)	France	Yes		Yes (Quantitative, by questionnaire and medical examination)			Patients from the free access to healthcare facility at the Baudelaire Hospital - Paris	536
Debono & Grazia (2016)	Malta		Yes (Report for the European Commission)	Desk analysis of national policies for asylum seekers and refugees				
Barda et al. (2016)	France)	Yes		Yes			The population of Médecins du Monde health centers in Paris and Saint Denis in particular vulnerable migrants who faced accomodation issue	
Dray Soira et al. (2015)	France	Yes		Yes			National data bases	778 outpatients in 20 healthcare settings
Bergeon & Hoyez (2015)	France	Yes				Yes	Ethnography and qualitative interviews to migrants living in squats	Well regarded study
Cognet et al. (2012)	France	Yes				Yes (qualitative interviews follow up sof the quantitative survey “Trajectoires et Origines”	Not newly arrived migrants but with a life course focus on discimination linked to the country of origin	
Bradby et al. (2015)	Europe	Yes (literature review)						
Tognetti (2015)	Italy	Yes (literature review)						
Affronti et al. (2014)	Italy	Yes (book chapter – desk research)						
Suess et al. (2014)	Spain	Yes (narrative review of comparative studies published between 2009 and 2012						
Cuadra (2010)	Europe		Yes (Comparative report or laws and regulations in Eu countries)				Survey among national experts in law and regulations on care for undocumented migrants	
Pithara et al. (2012)	Cyprus	Yes				Qualitative on temporary migrants to access and use effectively healthcare services in Cyprus	Semistructured interview with domestic workers and students	13 domestic workers and 13 students
Kouta et al. (2013)	Cyprus	Yes		Quantitative (closed-ended questionnaire referring to the knowledge, attitudes and behaviour of the participants in relation to HIV/AIDS				600 partecipants: migrant from non EU countries from two cities in Cyprus (Nicosia, Limassol).
Sanchez et al. (2016)	Cyprus		Yes (report)			Literature review and face to face interviews and focus group		9 immigrants from different countries
Marques (2012)	Europe		Yes (note reviewing undocumented migrants’ access to healthcare)					
Aditus (2013)	Malta		Yes (NGO report)	Standardized questionnaires			People in all phases of the settlement and integration processes in Malta.	156 people, 55 female, and 101 male.
Association Aides (2015)	France		Yes (NGO report)	Questionnaire	Yes		Migrants with heath issues. National foreign who apply to a regularization of their administrative situation in regards to their health status.	
Chappuis et al. 2015	France		Yes (NGO report)		Yes	Yes	Patients visiting Médecins du Monde (MdM) health centers in France	
Bas Sarmiento et al. (2015)	Spain (Campo de Gibraltar)		Yes			Qualitative		51 migrants from 11 countries
Kohls (2012)	Germany		Yes (report from the Federal Agency for Migration and Refugees)				Elderly people	
Frank et al. (2017)	Germany	Yes (literature review on the general health care delivery for refugees/asylum seekers)					Refugees/Asylum seekers	
Razum (2008)	Germany		Health reporting of the Federal German Government conducted by Robert-Koch-Institute and the Federal Statistical Office	Survey			Migrants	Statistically representative sample of the population
Taglieri et al. (2013)	Italy		Yes (Healthcare Institute report)	Report on the activity of the HIV phone counselling			Non-Italian population living in Italy and accessing the Intervention for the prevention of HIV infection	
Osservatorio della salute (2016)	Italy		Yes (National Institute of Statistic report)	Census data			Resident foreign population by municipality	Census
Grech & Pisani (2016)	Malta		NGO report			FGDs and in-depth interviews	Female population	9 Refugee Women from Syria, Somalia, Libya, Eritrea, Ethiopia, and Ivory Coast in Malta 2 health care providers
Råssjö (2013)	Sweden	Yes		Quantitative (retrospective case control study) - antenatal health record)			Migrants and non-migrants women using ante-natale care	523 Swedish-born women and 262 Somali women
Villadsen et al. (2016)	Europe; North America; Australia	Yes		Literature review and case study			Best practices Best Practice & Research Clinical Obstetrics and Gynaecoloy	
GLM study	Austria		Yes					
`Ganhal (2016)	Austria	Yes					Turkish, Bosnia/Croatia/Serbia	
Rosano (2015)	Italy and Spain	Yes		Quantitate, secondary analysis			Migrants and native population in Italy and Spain, Census data	European Union Statistics on Income and Living Conditions (EUSILC)
Koponen et al. (2014)	Finland	Yes		Yes –(Survey)			Migrants from Russian, Somali, Kurdish background aged 18–64 years	3000 persons of Russian, Somali or Kurdish origin
Wångdahl (2014)	Sweden	Yes		Yes (survey)			Adult refugees connected to language schools for migrants in Sweden	455 adult refugees
Crede´ et al. (2017)	Europe	Yes		Systematic literature review			International migrants’ using EDs in European Economic Area (EEA) countries compared with that of non-migrants	
Kohls (2011)	Germany	Yes		Statistical analysis			Foreign nationals	Data from the official statistics, data of the national central register of foreign nationals and of the statutory pension insurance
Halmdienst et al. (2013)	Europe		Yes		Longitudinal, multidisciplinary and international data collection (SHARE)		Migrants 50+, partly focus on groups from Former Yugoslavia	60,000 people over the age of 50 are examined for health, age, pension-specific and financial issues in around 20 European countries and Israel.
Reinprecht et al. (2016)	Austria (City of Vienna)		Yes	Survey		Qualitative interviews	Older migrant from Turkey, Former Yugoslavia, Poland, Iran	60,000 people over the age of 50 11 qualitative interviews
Tsitsakis (2017)	Greece	Yes		Secondary quantitative analysis			Data from five of the six public hospitals in the specified region; per clinic cross tabulation analysis of admission diagnosis and citizenship variables	
Ben Cheikh (2011)	Spain	Yes			Retrospective longitudinal study		2004–2007 Hospital discharges of the foreign population in public hospitals in Aragon	
Carmona at al. (2014)	Spain	Yes (Systematic literature review of survey-based evidence on the use of care among migrants)					Migrants	
Gistau et al. (2012)	Spain	Yes				Qualitative FGs and semi-structured interviews	Respondents of 4 professional profiles: directors or coordinators, physicians, nurses, and cultural mediators.	73 hospital and primary health care professionals
Psarros et al. (2016)	Greece	Yes				Action research to provide psychological support through education, training and psychological support	Caregivers who worked as volunteers in non-governmental organisations in the hotspot of Moria on the island of Mytilene and in Idomeni (near the northern Greek border)	57 caregivers (30 women and 27 men) with a mean age of 32·3 years, who worked continuously for 70 days on average.
Hunter (2016)	Europe	Yes (Commentary on healthcare for migrants and refugees in different European countries in historical perspective)					Migrants and Refugees	
Dara et al. (2016)	Europe	Yes		Yes A questionnaire investigating screening and management practices among refugees			National TB programme representatives of all EU/European Economic Area countries of the WHO European Region, Switzerland and six additional (Albania, Bosnia and Herzegovina, the former Yugoslav Republic of Macedonia, Montenegro, Serbia and Turkey	36 national TB programme representatives of low and intermediate TB incidence European countries/territories of the WHO European Region
Morgan (2016)	Greece	Yes (Interview with senior medical officer published in The Lancet)					Refugees in a Northern Greek camp	
Psoinos et al. (2017)	Greece	Missing from Zotero						
**Associazione Nazionale Comuni Italiani (Anci) et al. (2016)**	Italy		Yes NGO report			Overview of the literature an best practices on mental health of migrants and refugees in Italy	Young and adult asylum seekers and refugees	
Medici senza Frontiere (2016)	Italy		Yes NGO report	Survey		Qualitative (interview and focus group)	Mental health service providers and migrants with mental health problems	135 participants (providers and migrants)
HCDCP et al. (2016)	Italy, Greece and Spain		Yes NGO report			Report on health assistance to migrants, refugees and asylum seekers through civil society organizations.	Refugees and asylum seekers, but also internal migrants, irregular migrants, trafficked persons, internally displaced people	Representatives from civil society organizations and also representatives from public
Tsiamis, Riza et al. (2016)	Greece	Yes (correspondence on the Lancet)						
Alcaraz et al. (2014)	Spain	Yes		Cross-sectional study			Immigrant women	339 episodes of care from February 2008 to October 2011 in Valencia

## Data Availability

The data and material in the form of articles and reports are stored in a Zotero Group that belongs to the MigHealthcare Consortium and will not be shared. The Zotero Group is private to members of the MigHealthcare consortium. Access can only be provided if the leader of the project consents. A request for permission to access can be sent to the leader of the project: Pania Karnaki (p.karnaki@prolepsis.gr), Prolepsis, Institute of Preventive Medicine Environmental & Occupational Health 7, Fragoklisias street, 151 25, Marousi, Greece.

## Abbreviations

EU: European Union
FGC: Female Genital Circumcision
JRS: Jesuit Refugee Service
HIV/AIDS: Human Immunodeficiency Virus/Acquired Immunodeficiency Syndrome
LTBI: Latent Tuberculosis Infection
NGO: Non-Governmental Organization
OECD: Organization for Economic Cooperation and Development
PTSD: Post-Traumatic Stress Disorder
UN: United Nations
WHO: World Health Organization

## Footnotes

1 The MigHealthCare consortium includes 14 partners from 10 European countries among which universities, Non-Governmental Organisations (NGOS) and national authorities with a composite range of expertise on health, migration, equality and integration issues.
